# *Catunaregam spinosa* (Thunb.) Tirveng: A Review of Traditional Uses, Phytochemistry, Pharmacological Activities, and Toxicological Aspects

**DOI:** 10.1155/2021/3257732

**Published:** 2021-08-25

**Authors:** Deepak Timalsina, Hari Prasad Devkota, Deepti Bhusal, Khaga Raj Sharma

**Affiliations:** ^1^Central Department of Chemistry, Tribhuvan University, Kirtipur, Kathmandu 44618, Nepal; ^2^Graduate School of Pharmaceutical Sciences, Kumamoto University, 5-1 Oe-honmachi, Chuo-Ku, Kumamoto 862-0973, Japan; ^3^Program for Leading Graduate Schools, HIGO Program, Kumamoto University, 5-1 Oe-honmachi, Chuo-Ku, Kumamoto 862-0973, Japan

## Abstract

*Catunaregam spinosa* (Thunb.) Tirveng. (Syn. *Randia dumetorum* (Retz.) Lam.), belonging to the Rubiaceae family, is distributed in south Asian countries. It is used as a traditional medicine to treat gastrointestinal and hepatic problems and as an anti-inflammatory and antimicrobial agent. The main aim of this review is to collect and analyze the available scientific information on traditional uses, phytochemistry, and pharmacological activities of *C. spinosa.* The scientific information related to *C. spinosa* was collected from various resources and databases such as SciFinder, Scopus, PubMed, and other databases. *C. spinosa* was found to be an important crude drug of the traditional medicinal systems such as Ayurveda. It was found to be used by the people of India as an alternative medicine, while the fruit of this plant was found to be used in dietary regimens as well. Active phytochemicals such as catunarosides, randianin, and several other saponins and triterpenoids possess various pharmacological activities such as anti-inflammatory, hepatoprotective, antibacterial, and immunomodulatory activities. Many studies have been performed to isolate the active compounds; however, there is a need for more activity-guided isolation studies. Various in vitro studies showed promising results but there are not many studies related to mechanism of actions using animal models. Hence, future studies on *C. spinosa* should focus on correlating the traditional uses with active phytoconstituents and modern pharmacological activities.

## 1. Introduction

Humans have been dependent on plants since the ancient times for various reasons such as food, shelter, and medicine [1]. Medicinal plants are the primary source of health benefits in various communities of the world [2]. More than 80% of the world's population is reported to use plant-based medicines as primary healthcare as they are easily available and safe to use [3]. Recently, researchers have developed traditional knowledge about medicinal plants which can fulfill the current gap in therapeutics, nutraceuticals, drug discovery, and development [4]. Especially in developing countries, the reliance on plant medicine is a typical basis for addressing the ethnomedicinal knowledge in therapeutics from different ethnic communities [5].

**Figure 1:**
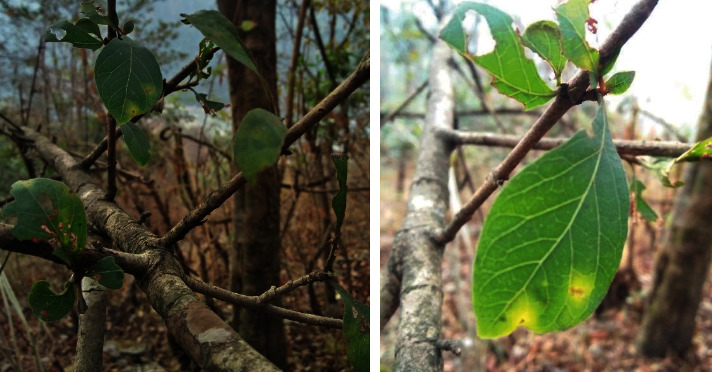
Photographs of *Catunaregam spinosa* (Thunb.) Tirveng.

Altogether 68 names of species are recorded under the genus *Catunaregam* belonging to family Rubiaceae [6]. The particular species *Catunaregam spinosa* (Thunb.) Tirveng. (Figure 1) is a shrub with thorns which is distributed up to 4000 ft. from the sea level [7]. The other synonyms of this species are given in Table 1 [6]. *C. spinosa*, known as Madana in Sanskrit, Madanphal in Nepali, and emetic nut or mountain pomegranate in English, is a deciduous shrub with approx. 5 m of height, leaves are ovate, simple, shiny, and pubescent [8], and flowers are white solitary and possess a honey-like fragrance [9]. This plant has been reported from various parts of India [10], Nepal, Bangladesh, South China [11], and the African subcontinent [12]. *C. spinosa* is reported to be distributed in tidelands of semitropical and subtropical areas [13]. The ancient medicinal systems of Ayurveda and Siddha use this plant to treat various types of symptoms and it is still in use [14]. “Rasayana,” a kind of clinical specialties in Ayurveda, uses this plant for promoting good habits in the dietary regimen [15]. People in India and Brazil use this plant for treating food poisoning, inducing vomiting, and treating allergy and inflammation [16]. The presence of various important phytoconstituents such as phenols, flavonoids [17], triterpene saponins [18, 19], hemolytic saponins, randianin [20], iridoids [21], dihydroisocoumarin [13], and many more other phytoconstituents is reported from various parts. Several studies have reported the modern pharmaceutical activities of *C. spinosa* such as piscicidal [22], molluscicidal [23], antioxidant [8], anti-inflammatory, antidiabetic, and antihyperlipidemic [24] activities. The isolated compounds are not well studied for the possibility of drug discovery pathways. It is important to have a clear idea of this medicinally important plant and its scientific progress. The review of the current state of *C. spinosa* and its overall perspective related to ethnomedicinal importance and modern medicine is still lacking. Thus, this review aims to collect and analyze the current status of traditional uses, phytochemicals, and pharmacological activities from the available scientific information.

## 2. Methodology

The scientific information of *C. spinosa* was collected from various online databases such as SciFinder, Scopus, Google Scholar, PubMed, Web of Science, and ScienceDirect. Additionally, the books, proceedings, comments, and editorials were used as secondary sources. The scientific names and their synonyms were cross-checked from the database available in the website of “The Plant List” (http://www.theplantlist.org/). The articles of better quality with sufficient taxonomical, ethnomedicinal, and pharmacological information were selected while preparing this review.

## 3. Traditional Uses in Medicine and Food

The fruit of *C. spinosa* is traditionally used to treat various symptoms (Table 2). Almost all parts of the plant have been used as a traditional medicine in Ayurveda and fruits have been reported to be used in medicine as well as in food. This plant is reported to treat ulcers, inflammation, tumors, wounds, and skin diseases [7]. People from the upper hilly region of India used its bark for curing diarrhea and dysentery [25]. The fruits or pulps of this plant are used as an abortifacient [26] and nauseant [11], as well as for treating asthma [27, 28]. The fruit of *C. spinosa* is also taken as a nutrient or diet.

## 4. Chemical Constituents

**Figure 2:**
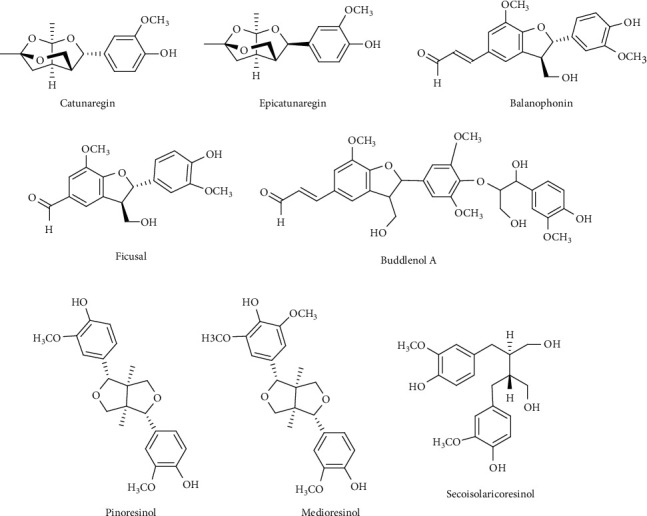
Structures of lignans.

**Figure 3:**
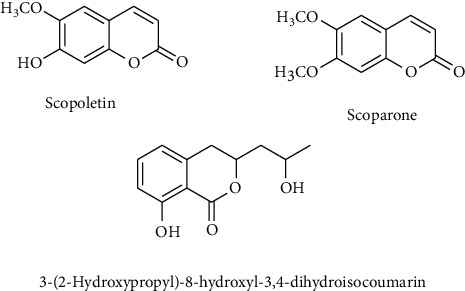
Structure of coumarins and isocoumarin.

**Figure 4:**
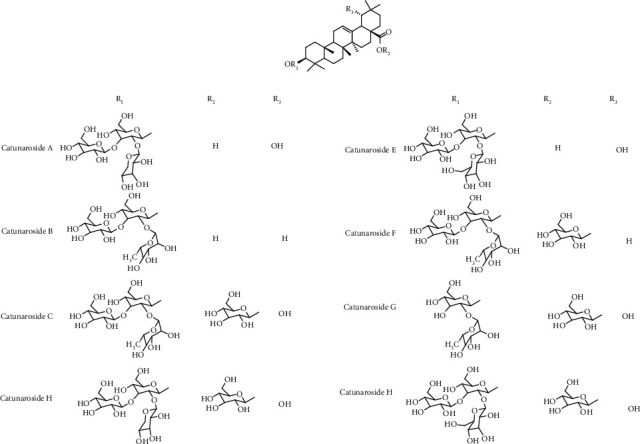
Structures of catunarosides A–H.

**Figure 5:**
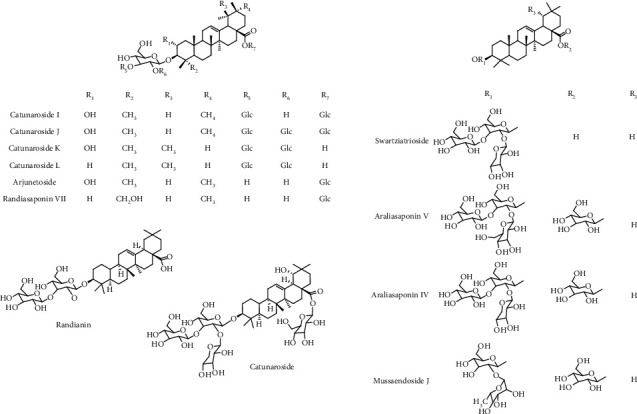
Structures of catunarosides I–L and other saponins.

**Figure 6:**
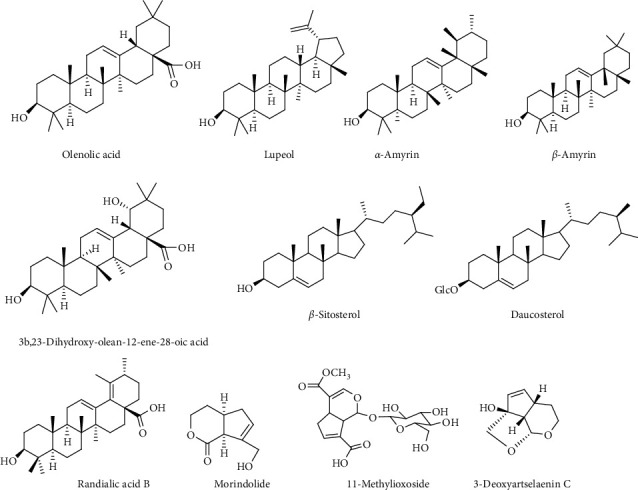
Structures of steroids, terpenoids, and other compounds.

Various flavonoids, alkaloids, tannins, lignans, terpenoids, and volatile oils have been reported from this plant. The triterpene, saponins [21], and iridoid glucosides were isolated [42] from the fruits of *C. spinosa*. Three triterpenoid saponins, 3-O-[*β*-D-glucopyranosyl-(1⟶3)-*β*-D-6-O-methyl-glucuronopyranosyl oxy]-2*β*-hydroxy-olean-12-en-28-oic acid, 3-O-[*β*-D-glucopyranosyl-(1⟶2)-*β*-D-glucopyranosyl]-olean-12-en-28-oic acid, and 3-O-[*β*-L-rhamnopyranosyl-(1⟶3)-*β*-D-glucuronopyranosyl-(1⟶2)-*β*-D-glucopyranosyl-(1⟶2)-*β*-D-glucopyranosyl]-12-en-28-oic acid, and two triterpenoids, oleanolic acid and 3*β*,23-dihydroxy-olean-12-ene-28-oic acid, were isolated from fruits [43]. From the stem bark of *C. spinosa*, the compounds named catunaregin and epicatunaregin were isolated [44]. Many triterpenoid saponins such as catunarosides were reported [45] from the n-BuOH extract of the stem bark of this plant. There have also been studies on nutritional contents of the fresh fruit of this plant and they were reported to contain a high amount of carbohydrate [38]. 11-Methylixoside was isolated from the bark of *C. spinosa* [46]. The bioactive compounds are enlisted in Table 3 and their structures are in Figures 2–6.

## 5. Pharmacological Activities

Due to the widespread traditional uses, *C. spinosa* extracts and compounds have been subjected to various pharmacological activity evaluations. This plant has been studied widely for its pharmacological activities, mainly antibacterial, antioxidant, anti-inflammatory, antimicrobial, anticancer, and antifertility activities. Few studies have reported sedative and analgesic activities. Most of the activities are based on random screening rather than traditional knowledge, which cannot provide the correlation between ethnomedicine and modern pharmacological activities. Some studies carried out on *C. spinosa* are discussed in the following section.

### 5.1. Antimicrobial Activities

The extract obtained from the fruit bark of *C. spinosa* showed prominent antibacterial activity against various species of *Proteus, Staphylococcus, Clostridium, Salmonella, Vibrio, Bacillus, Escherichia,* and *Pseudomonas* [53].

The water extract of the seed of *C. spinosa* was tested against human pathogenic bacteria such as *Staphylococcus aureus*, *Bacillus subtilis*, *Escherichia coli*, *Klebsiella pneumonia,* and *Proteus mirabilis*; and the minimum inhibitory concentration (MIC) was found to be 0.07, 0.08, 0.88, 3, and 0.73 mg/mL, respectively, and the zone of inhibition ranged from 15 to 20 mm [36].

In another study, the antimicrobial activity was performed by the broth dilution method and disc diffusion method against various Gram-positive and Gram-negative bacteria. The methanolic extract of leaves of *C. spinosa* showed a potent effect against Gram-negative bacteria, that is, *K. pneumoniae*, *E. coli,* and *S. typhi*, as compared to Gram-positive bacteria, that is, *B. subtilis* and *S. aureus* [40].

The fruit extract of *C. spinosa* was subjected to its antimicrobial activity by the disc diffusion method, where it was tested against *P*. *aeruginosa*, *E. coli*, *S. pyogenes*, *S. typhi*, *S. pyogenes*, and *B. subtilis*. The activity of the extract was tested as compared to the standard Chloramphenicol. One hundred *µ*g/mL of the ethanolic extract showed more potency and highest zone of inhibition of 28.0 mm against the *Bacillus* and *Klebsiella pneumoniae* of 24.0 mm and the lowest was that of *Streptococcus pyogenes*. In chloroform extract, the maximum zone of inhibition of 26 mm was recorded for *Bacillus subtilis* followed by *Pseudomonas aeruginosa* of 16 mm and it was lower for the remaining bacteria. In the aqueous extract, the maximum zone of inhibition of 18 mm was recorded for *Klebsiella pneumonia* followed by *Bacillus subtilis* of 14.0 mm and it was lower for other bacteria [54].

### 5.2. Hepatoprotective Activities

The hepatoprotective potential of methanolic extract of the fruit of *C. spinosa* at the dose of 200 mg/kg was studied in male albino Wistar rats with alcohol (28.5%) induced liver injury. The biochemical parameter and histopathology of the liver were accessed before and after treatment and were found to be significant to retrieve the parameters towards the normal stage. The treatment with standard silymarin and *C. spinosa* extract showed a decrease in the elevated levels of alanine aminotransferase (ALT), aspartate aminotransferase (AST), direct bilirubin (DB), and triglyceride (TG) as compared to rats exposed to alcohol only. The histopathological examination showed severe necrosis in cells treated with alcohol only, while reduced necrosis was found in the rats treated with *C. spinosa* extracts [55].

A similar study was performed in carbon tetrachloride (CCl_4_) induced hepatic damaged rats [17]. The animal treated with *C. spinosa* leaf and bark extracts at 400 mg/kg for 15 days showed a significant decrease in various biochemical parameters such as ALP, AST, ALT, LDH, albumin, and total and direct bilirubin and an increase in total protein levels as compared to the CCl_4_ only treated group.

However, in these studies, the mechanism by which *C. spinosa* is protecting liver injury is yet to be studied. Further, the studies on the isolation and identification of active compounds and elucidation of their mechanism of action are necessary.

### 5.3. Antioxidant Activities

Methanolic extracts obtained from the fruit bark of *C. spinosa* were studied for their antioxidant activities. The DPPH radical scavenging assay, nitric-oxide scavenging assay, and superoxide inhibition assay were done for which the value was expressed in EC_50_. The EC_50_ value for the DPPH assay for the extract was 37 *µ*g/mL, whereas the EC_50_ value for standard (pyrogallol) was 1.7 *µ*g/mL. The EC_50_ value of the extract for nitric-oxide scavenging assay was 16.8 *µ*g/mL as compared to the standard (curcumin: 11.2 *µ*g/mL). Similarly, the EC_50_ value of the extract for superoxide anion activity was 8.4 *µ*g/mL compared with the standard (ascorbic acid: 12.5 *µ*g/mL) [53].

In another study to evaluate the DPPH free radical scavenging activity for the methanolic extract of leaves, the extract showed the IC_50_ values of 45.12 *µ*g/mL as compared to ascorbic acid (34.91 *µ*g/mL). Similarly, in the ferrous ion chelating activity study, the extract had the IC_50_ value of 64.70 *µ*g/mL as compared to ascorbic acid (30.96 *µ*g/mL) [56].

The DPPH and ABTS ^+^ scavenging assay were performed to determine the antioxidant potential of leaves of *C. spinosa* [40] . The IC_50_ value recorded was 0.154 ± 0.14 and 0.175 ± 0.12 mg/mL, respectively. Another study recorded the IC_50_ value of stem and leaves of *C. spinosa* as 8.08 and 5.35 mg/mL, respectively, as compared to the IC_50_ value of ascorbic acid (<2 mg/mL) [39].

All these antioxidant activity evaluation studies were based on the *in vitro* analysis. The *in vivo* antioxidant properties and their mechanism of action are yet to be studied.

### 5.4. Anti-Inflammatory Activities

Anti-inflammatory activities of *C. spinosa* were studied in the carrageenan-induced inflammation test in the hind paw of rats. The inflammation was inhibited by 31.69, 35.11, and 41.62% at 1, 3, and 5 h, respectively, by 200 mg/kg of a dose of ethanolic extract of leaves of *C. spinosa*, which was comparable to the inhibition of standard drug indomethacin (10 mg/kg) [57].

### 5.5. Antihyperglycemic Activities

Antihyperglycemic activities of methanolic extract of *C. spinosa* fruit extract were studied in the streptozotocin-induced male Wistar diabetic rats. The group of rats treated with standard drug gliclazide and *C. spinosa* fruit extract showed a decreased level of glucose as compared to the diabetic control group. The value of blood sugar decreased from 88.5 ± 6.86 to 74.16 ± 4.02 in gliclazide (50 mg/kg) treated group in between 60 and 120 min, while *C. spinosa* fruit extract (400 mg/kg) decreased the blood sugar level from 106.16 ± 7.8 to 87.5 ± 5.00 mg/dL [24]. The detailed biochemical mechanism by which it acts against hyperglycemia is yet to be studied. The study of isolation of bioactive compounds which can act as hypoglycemic activity from *C. spinosa* and their characterization can lead to the development of potential drugs in the future.

### 5.6. Wound Healing Activities

The wound healing was performed in the HUVECs cell and it was treated with catunaregin obtained from the plant *C. spinosa* at concentrations of 10, 50, and 100 *μ*M and found to be significant for preventing cell migration. A maximum decrease of 65.4% in cell migration was recorded for 100 *μ*M. The next invasion assay was done to demonstrate the antiangiogenesis activity. In this assay, catunaregin significantly decreased the invasion of HUVECs induced by VEGF. The highest decrease of the invasion of HUVECs (61.3%) was recorded for the concentration of 100 *μ*M [31].

### 5.7. Anticataleptic Activities

The anticataleptic efficiency of the ethanolic extract of fruits of *C. spinosa* was determined in the catalepsy in mice induced by clonidine and haloperidol. In this study, the forepaw of mice was placed in the bar and the time required to remove the paw from the bar was recorded. The catalepsy recorded for clonidine was 229.2 ± 14.88 sec., which was significantly decreased to 135.2 ± 4.84 seconds when treated with 720 mg/kg of ethanolic extract of fruits of *C. spinosa* similar to that of standard chlorpheniramine maleate [58].

### 5.8. Other Activities

The anthelmintic activity of the fruit extract of *C. spinosa* was studied in *Pheretima posthuma*. The extract of *C. spinosa* (200 mg/mL) was tested against worms for its ability to paralyze and kill the worms. The study showed that the ethanolic extract revels comparable results similar to albendazole (10 mg/mL), a standard drug as a positive control [59].

There are several other ethnomedicinal practices of *C. spinosa* such as treating ulcers, asthma, abortifacient, curing piles, jaundice, and others on which no pharmacological studies have been made. Further, research can be done on the wide range of pharmaceutical activities related to its ethnomedicinal value.

## 6. Toxicological Aspects

Acute toxicity study showed that the aqueous and alcoholic extracts of *C. spinosa* at a dose of 2000 mg/kg in a Swiss albino mice model were found to be nonlethal [14]. Another study regarding the toxicity of 11-methylioxoside obtained from the bark of *C. spinosa* was reported by Jangwan et al. [46]. Upon administrating the single dose of 5000 mg/kg extract of *C. spinosa* in Wistar albino rats, no mortality was observed. However, when administrating the different doses of 200 mg/kg, 2000 mg/kg, and 5000 mg/kg continuously up to 14 days, the mortality observed was 0%, 25%, and 50%, respectively. The result was supported by a change in biochemical parameters and histopathology reports [60].

Brine shrimp lethality assay was done to study the cytotoxic activities of *C. spinosa* leaf extract and lethal concentration (LC_50_) was determined. The LC_50_ values of water and ethanol extract were 0.94 and 0.23 *µ*g/ml, respectively [12]. A similar study was done in the methanolic extract of the stem and the LC_50_ value was found to be 1.32 *µ*g/mL [29].

## 7. Conclusion and Future Prospects

*C. spinosa* is reported to be used in traditional medicine as well as in dietary regimens. Almost all parts of the plant have been used in traditional medicines such as leaves, fruits, stem, stem bark, fruit bark, and root bark. Fruits are also used in food. Various classes of compounds such as polyphenols, flavonoids, terpenoids, triterpenoids, and saponins are reported to be the main constituents found in this plant. The diverse pharmacological activities such as anti-inflammatory, antimicrobial, antidiabetic, antioxidant, and hepatoprotective activities have been investigated with promising results. Despite those promising results, many studies are based only on the *in vitro* models, and mechanisms of action are not well studied. Some of the research lacked the proper use of positive and negative control in the experiment setting. The *in vivo* studies were performed with very high doses of extracts and seem practically less efficient. Some ethnopharmacological reports show that this plant has been used to treat ulcer symptoms and acidity; however, pharmacological activities were not performed to evaluate these activities. Future research work must correlate the traditional knowledge with the isolation of active constituents and corresponding pharmacological significance. Along with this, the details of toxicological activities if studied can pave the way to develop new therapeutic agents.

## Figures and Tables

**Table 1:** Synonyms of *C. spinosa* [6].

*Canthium chinense* Pers.
*Canthium coronatum* Lam.
*Ceriscus malabaricus* Gaertn.
*Gardenia dumetorum* Retz.
*Gardenia dumosa* Salisb.
*Gardenia spinosa* Thunb.
*Gardenia stipularis* Rottler
*Genipa dumetorum* (Retz.) Baill.
*Narega coduva *Raf.
*Posoqueria dumetorum* (Retz.) Willd. ex Roxb.
*Posoqueria floribunda* Roxb.
*Randia floribunda* (Roxb.) DC.
*Randia lachnosiphonium* Hochst.
*Randia oxypetala* Lindl.
*Randia rottleri* Wight & Arn.
*Randia spinosa* (Thunb.) Poir.
*Randia stipulosa* Miq.
*Randia tomentosa* Wight & Arn.
*Randia uniflora* Regel
*Solena dumetorum* (Retz.) D.Dietr.
*Solena floribunda* (Roxb.) D.Dietr.
*Solena longispina* D.Dietr.
*Solena nutans* D.Dietr.
*Xeromphis retzii* Raf.

**Table 2:** The traditional uses of *C. spinosa* in medicine and food.

Country (locality if available)	Plant parts	Uses	References
Bangladesh	Fruits	Taken as a juice for treating anthelmintic, tonic, and curing piles, flatulence	[12, 29]
Bangladesh, (Bagerhat)	All parts	Taken in the form of juice for treating urinary tract infections and genital disorder	[30]
Brazil	All parts	Taken as a crushed paste for treating dysentery and inflammations	[13]
China (Hainan)	Stem bark	Taken in the form of paste to prevent the growth of cancer	[31]
India	Barks	Crushed bark juice is taken to treat diarrhea and dysentery	[25]
India	All parts	Taken in the form of juice in an empty stomach for improving the defense mechanism of the body and improving longevity	[32]
India	Leaves	Used in the form of a solution and is useful in controlling Molluscans	[23]
India (Assam, Manipur)	All parts	Taken in the form of juice for treating liver ailments	[17]
India (Kalakkad, Tamil Nadu)	Root bark and stem bark	Taken in the form of juice in an empty stomach for treating constipation, fever, and an antiseptic	[33]
India (Karnataka)	All parts	Apply in the form of paste as well as juice for treating various skin diseases	[34]
India (Kedarnath)	Fruits	Apply in the form of paste for treating skin disease, in the form of juice for ulcers, abortifacient, cough, rheumatism, and fever	[26, 35]
India (Kerala)	Seeds	The solution of crushed seeds in water is used in controlling mosquito larva	[36]
India (Kolli and Boda hills)	Fruits	Taken as a vegetable for vitamin and mineral content, nutritional diet	[37]
India (Nasik)	Flowers	Taken as vegetable for improving dietary habits	[38]
India (Pune, Goa)	Fruits	Taken in the form of juice in treating gonorrhea, asthma, tonic, emetic, jaundice, dysentery	[27, 39]
India (Sikkim, Bengal, Maharashtra)	Fruits	Applied in the form of paste in treating allergy, wound infections, and skin diseases	[10]
India (Western Ghats)	All parts	Taken as a juice for treating rheumatism, diarrhea, and viral diseases	[40]
Nepal (Chitwan)	Ripen fruits	Crushed seed and its solution is effective in treating fish poison	[22]
Sri Lanka	Bark and leaves	Apply in the form of paste for curing skeletal fractures	[41]

**Table 3:** Bioactive compounds reported from *C. spinosa*.

Chemical class	Compounds	Plant parts	References
Lignans	Catunaregin, epicatunaregin, balanophonin, ficusal, 5″-methoxy-4″-O-(8- guaiacylglycerol)buddlenol A, pinoresinol, medioresinol, secoisolariciresinol	Stem bark	[13, 31, 44]

Coumarins and isocoumarin	Scopoletin, scoparone, 3-(2-hydroxypropyl)-8-hydroxyl-3,4-dihydroisocoumarin	Stem bark	[13]

Steroids, triterpenoids, and their glycosides (saponins)	Catunaroside A, catunaroside B, catunaroside C, catunaroside D, catunaroside E, catunaroside F, catunaroside G, catunaroside H, catunaroside I, catunaroside J, catunaroside K, catunaroside L, Arjunetoside, Randiasaponin IV, swartziatrioside, araliasaponin V, araliasaponin IV, mussaendoside J, daucosterol, *β*-sitosterol, lupeol, 1-keto-3-hydroxyoleanane, randialic acid B	Stem bark	[9, 13, 19, 45, 47–50]
	Urosaponin, dumetorinins A-F, randianin, 3-O-[*β*-D-glucopyranosyl-(1⟶3)-*β*-D-6-O-methyl-glucuronopyranosyl oxy]-2*β*-hydroxy-olean-12-en-28-oic acid, 3-O-[*β*-D-glucopyranosyl-(1⟶2)-*β*-D-glucopyranosyl]-olean-12-en-28-oic acid, 3-O-[*β*-L-rhamnopyranosyl-(1⟶3)-*β*-d-glucuronopyranosyl-(1⟶2)-*β*-D-glucopyranosyl-(1⟶2)-*β*-D-glucopyranosyl]-12-en-28-oic acid, 3*β*,23-dihydroxy-olean-12-ene-28-oic acid and olenolic acid, 3-O-[O-*β*-D-glucopyranosyl-(1⟶4)-O-*β*-D-glucopyranosyl-(1⟶3)-(*β*-D-glucuronopyranosyl)]oleanolic acid, 3-O-[O-*β*-D-glucopyranosyl-(1⟶6)-O-*β*-D-glucopyranosyl-(1⟶3)-(*β*-D-glucuronopyranosyl)]oleanolic acid, randioside A	Fruits	[9, 18, 20, 43, 51, 52]
	1-Keto-3*α*-hydroxyoleanane, *α*-amyrin, *β*-amyrin	Root bark	[19]

Iridoids	11-Methylioxiside	Leaves	[9, 21, 46]
	3-Deoxyartselaenin C, morindolide	Stem bark	[13]

## Data Availability

Data sharing is not applicable. No new data were generated.

## Abbreviations

ALT: Alanine aminotransferase
AST: Aspartate aminotransferase
DB: Direct bilirubin
DPPH: 1,1-Diphenyl-2-picrylhydrazyl
HbA_1_C: Glycosylated hemoglobin
HUVECs: Human umbilical vein endothelium cells
IC_50_: Inhibition concentration 50%
LC_50_: Lethal concentration 50%
MIC: Minimum inhibitory concentration
TB: Total bilirubin
TG: Triglyceride
ZOI: Zone of inhibition.
